# Use of indices to measure socio-economic status (SES) in South-Asian urban health studies: a scoping review

**DOI:** 10.1186/s13643-018-0867-6

**Published:** 2018-11-17

**Authors:** K. M. Saif-Ur-Rahman, Iqbal Anwar, Md. Hasan, Shahed Hossain, Sohana Shafique, Fariha Haseen, Md. Khalequzzaman, Aminur Rahman, Shariful Islam

**Affiliations:** 1 Health Systems and Population Studies Division, icddr,b , Dhaka, Bangladesh; 2 Department of Public Health and Informatics, Bangabandhu Sheikh Mujib Medical University (BSMMU), Dhaka, Bangladesh; 3 Systematic Review Centre (SRC), Bangabandhu Sheikh Mujib Medical University (BSMMU), Dhaka, Bangladesh

**Keywords:** Inequality, Urban poor, Urban health, Health research, Indicators, Socioeconomic status, Scoping review, Systematic review, South Asia

## Abstract

**Background:**

Universal health coverage (UHC) is a key area in post-2015 global agenda which has been incorporated as target for achieving health-related Sustainable Development Goals (SDGs). A global framework has been developed to monitor SDG indicators disaggregated by socioeconomic and demographic markers. This review identifies the indices used to measure socio-economic status (SES) in South Asian urban health studies.

**Methods:**

Two reviewers searched six databases including Cochran Library, Medline, LILACS, Web of Science, Science Direct, and Lancet journals independently. All South Asian health studies covering urban population, with any research-designs, written in English language, and published between January 2000 and June 2016 were included. Two reviewers independently screened and assessed for selection of eligible articles for inclusion. Any conflict between the reviewers was resolved by a third reviewer.

**Results:**

We retrieved 3529 studies through initial search. Through screening and applying inclusion and exclusion criteria, this review finally included 256 articles for full-text review. A total of 25 different SES indices were identified. SES indices were further categorized into 5 major groups, e.g., (1) asset-based wealth index, (2) wealth index combining education, (3) indices based on income and expenditure, (4) indices based on education and occupation, and (5) “indices without description.” The largest proportion of studies, irrespective of country of origin, thematic area, and study design, used asset-based wealth index (*n* = 142, 54%) as inequality markers followed by the index based on income and expenditure (*n* = 80, 30%). Sri Lankan studies used income- and expenditure-based indices more than asset-based wealth index. Majority of the reviewed studies were on “maternal, neonatal, and child health” (*n* = 98, 38%) or on “non-communicable diseases” (*n* = 84, 33%). Reviewed studies were mostly from India (*n* = 145, 57%), Bangladesh (*n* = 42, 16%), and Pakistan (*n* = 27, 11%). Among the reviewed articles, 55% (*n* = 140) used primary data while the rest 45% studies used secondary data.

**Conclusion:**

This scoping review identifies asset-based wealth index as the most frequently used indices for measuring socioeconomic status in South Asian urban health studies. This review also provides a clear idea about the use of other indices for the measurement SES in the region.

**Electronic supplementary material:**

The online version of this article (10.1186/s13643-018-0867-6) contains supplementary material, which is available to authorized users.

## Background

The tenacity towards achieving universal health coverage (UHC) is central to the post-2015 global agenda which commits “leaving no one behind” and is incorporated as a target in the Sustainable Development Goals (SDGs) [1]. A global framework has been developed to track progress in SDG indicators disaggregated by socioeconomic and demographic strata in order to allow assessment of the equitable distribution and financial risk protection [2]. The unbiased measurement of socio-economic status (SES) is crucial for such benefit-incidence analysis in health, population, and nutrition. Literature suggests that SES has diverse definitions and multiple ways to measure [3]. Conventionally, income is a core SES indicator and some SES measures are solely based on per capita income such as “Prasad’s scale” [4]. Considering high level of unreliability [3], including the unwillingness of people to discuss about income, social scientists consider “consumption” or “expenditure” as better markers of SES than income [5]. Composite SES indices are used that usually incorporate education and occupation along with income to reflect three distinct and interrelated dimensions of class, status, and power of social hierarchy [6]. Others preferred “education” or “occupation” as proxy for SES. The problems with such proxy measures are that they divide population into unequal-sized groups making causal interpretations difficult [5]. However, all SES indices commonly used in epidemiological studies have their own strengths and weaknesses [3]. Researchers are working hard to identify suitable SES indices to measure inequality in different contexts. Several tools are now available with multiple combinations of component indices to assess SES in different contexts. For example, Kuppuswami et al. [7] has combined material possession, education, occupation, and income in his composite SES scale; Pareekh et al. [8] added caste and family type and created a new scale with a total of nine indicators; and Tiwari et al. [9] used seven profiles (housing, material possession, education, occupation, economic profile, cultivated land, and social profile) in his scale. In *Gour’s classification* (2013), expenditure is combined with income, education, occupation, and living standard [10]. A similar SES scale has been proposed by Bhuiya et al. [11] for rural Bangladeshi population where social involvement, food, clothing, education, shelter, and health were incorporated in the composite SES indicator. Other indices such as Multidimensional Poverty Index (MPI) and unsatisfied basic needs (UBN), which are based on different economic theories, are capable of identifying non-income factors associated with social inequalities [12].

A newer and more objective way of measuring SES is wealth index (WI) where construction materials of dwelling houses and household assets are combined together through data reduction using statistical procedure of principal component and factor analysis (PCA & FA) methods to come up with a summary WI (usually in quintiles). Related asset information is usually extracted from household survey or census data. Results from validation study [13] showed almost the same interpretation as the SES index constructed from income, consumption, or expenditure [13]. The WI [5] is thus a composite and relative measure of households’ SES [3]. The WI has been constructed from national household surveys such as Demographic Health Surveys (DHSs) in 56 countries and the National Family and Health Survey (NFHS) in India [12]. Although the method of choosing component variables is not well defined [14], experts opined that context-specific WI is a useful tool for measuring inequalities and widely used in low and middle income settings [12]. In this scooping review, we attempt to identify a range of indices used to measure SES in epidemiological studies in South Asian urban countries covering urban population.

## Methods

This is a scoping review to identify different indices used to measure SES in South Asian urban health studies.

### Types of studies

All epidemiological studies with use of any socioeconomic indices as an explanatory, outcome, or confounding variable were included in this scooping review.

### Population

All eligible studies conducted in South Asian countries (as defined by the World Bank) [15] covering urban population were included.

### Types of interventions

No specific intervention was targeted; rather, all studies including observational studies using different SES indices were considered.

### Outcome measures

All health-related studies using SES indices were targeted.

### Inclusion and exclusion criteria

All South Asian urban health studies, using any socioeconomic indices, and published in English language between January 2000 and June 2016 were included. Studies covering rural population in addition to urban dwellers were also considered. Any research design, without any restrictions on sample size, was allowed. Studies without the use of SES indices, conducted outside South Asian region, without urban population, published in other language (than English), beyond human health, and conducted beyond the mentioned time period (Jan 2000–June 2016) were excluded. Gray literature and unpublished works were excluded.

### Data sources and literature search

We searched six electronic databases: Medline (through PubMed), the Cochrane Databases, Science Direct, the Web of Science, LILACS, and the Lancet Series to retrieve relevant articles. We used the following key search terms for population, intervention, comparison, and outcome (PICO) (Table 1).

**Table 1:** Key terms used for developing comprehensive search strategy

Population (P)	Intervention (I)	Outcome (O)	Filter
Poor Poverty Urban Metropolitan Town^*^ Local government Local authority	Wealth Index Quintiles Status Condition Asset Socioeconomic Social Factors “Poverty index” Inequality Disparity	Health	South Asia Afghanistan Bangladesh Bhutan India Nepal Pakistan Sri Lanka Maldives

We developed a comprehensive and contextualized search strategy for each of the databases using key terms and database-specific index terms (see Additional file 1). Endnote software (version 7.0) was used for database management including duplication checking while EPPI reviewer software (version 4.6.0.1) was used for screening purposes.

### Screening process

Two reviewers (KMSUR and MH) independently screened the title and the abstract of each included article based on a set of codes for inclusion and exclusion criteria. After screening titles and abstracts, full texts of included articles were screened using the same set of codes for inclusion and exclusion. Any disagreements between the two reviewers were resolved by the third reviewer (SH).

### Data extraction and analysis

We developed a standard template to capture relevant aspects of the review objective. The template comprised of descriptive characteristics of the included studies such as author(s), year of publication, study design, analysis type, data source, geographic location, study theme, indices used to measure socioeconomic status, and types of population targeted (urban and/or rural). Extracted data were analyzed to address the review objectives. The obtained SES indices were categorized according to the similarity of component variables, formulation process, and their combinations (Table 2).

**Table 2:** Different category of available indices found in the scoping review

Measurement indices (ingredients used)	Name of the indices	Frequency (*n* = 265)	Percentage
Asset-based wealth index (using PCA & FA methods)	Wealth index (WI)	109	142 (54%)
	Standard living index	20	
	Socio-economic status (SES)	5	
	Economic status	4	
	Living condition	1	
	Living index	1	
	Poverty score	1	
	SES by factor analysis	1	
Wealth index combining education	Modified Kuppuswami classification	10	21 (8%)
	Socioeconomic status scale	3	
	Socioeconomic index	2	
	Multidimensional Poverty Index (MPI)	2	
	Unsatisfied basic needs (UBN)	2	
	Social Gradient Score	1	
	Kutty’s classification	1	
Indices based on income and expenditure	Income	77	80 (30%)
	Modified BG Prasad classification	2	
	Expenditure	1	
Indices bases on occupation and education	Types of school	8	16 (6%)
	Social status index	2	
	Occupation	2	
	Occupation and education	1	
	Occupation, education, household utility	1	
	Socio-economic class	1	
	Socioeconomic status based on education, Occupation and SE scale	1	
Indices without any description	Used indices without description and reference	6	6 (2%)

## Results

**Fig. 1:**
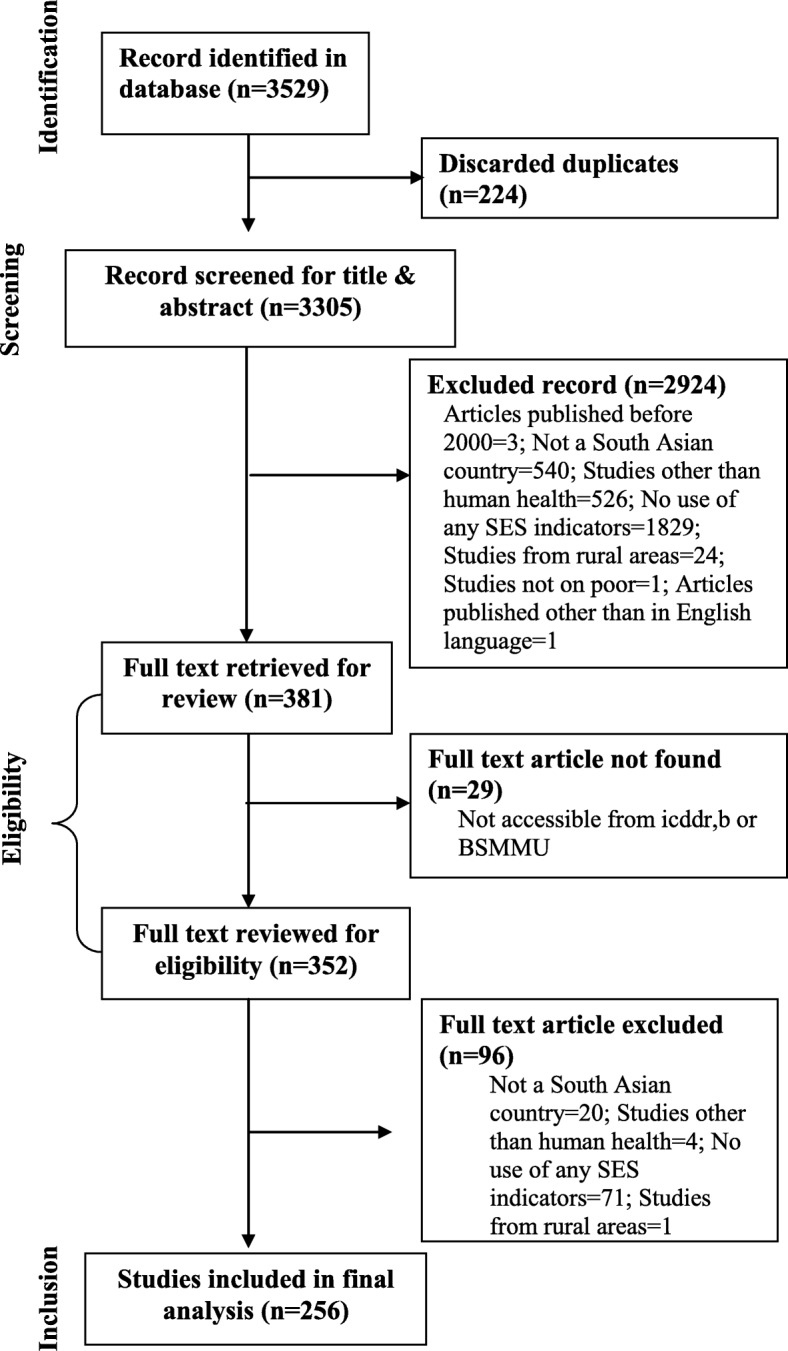
Scoping review flow diagram using PRISMA

Initial search yielded 3529 results of which 224 articles were discarded for duplication. The titles and abstracts of the remaining 3305 articles were screened applying the inclusion and exclusion criteria, and through this process, more 2924 articles were excluded, as they failed to meet the inclusion criteria, and 381 articles were selected for full-text review. Reviewing the full texts of these 381 articles, we identified 256 articles for the final analysis. A detailed description of the selection process is given in the PRISMA flow diagram (Fig. 1).

The scoping review identified the urban health studies in South Asian region between January 2000 and June 2016 with the use of 25 different types of SES indices (Table 2). Detailed descriptions of these indices are provided in Additional file 2. For better understanding, we further divided these 25 SES indices in 5 major categories based on underlying approaches, ingredients, and their different combinations.

**Fig. 2:**
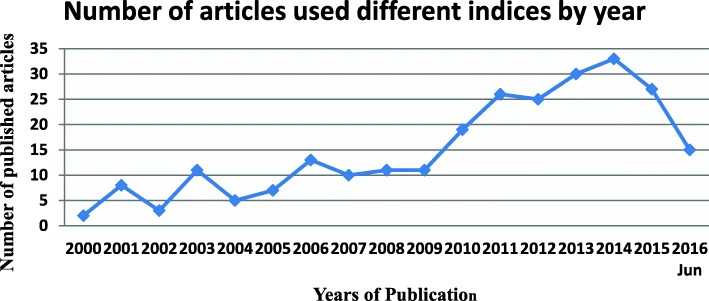
Number of articles used different indices by year (*n* = 256)

Asset-based wealth index was the most frequently used SES indicator irrespective of year of publication, country of origin, or thematic area of the study. Uses of other SES indices were minimum and some with even single frequency (Table 2). Fewer number of studies using SES indices were available during the first 5 years of the study period (*n* = 29, 11%), number of studies using SES indices increased gradually over time, and the majority of studies included in the review were published after 2010 (*n* = 175, 68%). The highest number of articles with SES indices was published during year 2014 (*n* = 33, 13%) (Fig. 2).

Table 2 shows the distribution of studies by types of SES indices used with their ingredients (or their combinations) and methods used. The largest proportion of articles used asset-based wealth index (*n* = 140, 54%) followed by indices based on income and expenditure (*n* = 80, 30%). A number of studies used wealth index combining education (*n* = 21, 8%), while fewer studies used indices based on occupation and education (*n* = 16, 6%). It was not possible to classify six articles (2%) to any of the abovementioned groups due to ambiguous description of use of indices and was classified as “indices without any description.”

Majority of the studies included in the review were from India (*n* = 145, 57%), followed by Bangladesh (*n* = 42, 16%) and Pakistan (*n* = 27, 11%) (Table 3). Fewer number of studies were from Nepal (*n* = 14, 5%) and Sri Lanka (*n* = 10, 4%), and only one was from Afghanistan. There was no published article from Maldives and Bhutan satisfying inclusion and exclusion criteria. Majority of included studies were descriptive in nature (*n* = 228, 89%). Fifty-five percent of included studies were from primary data while the rest 45% were secondary data analysis studies.

**Table 3:** Characteristics of included studies based on design, geographical distribution, types of data and theme

Characteristics	Frequency (*n* = 256)	Percentage
A. Study design		
Cross sectional study	228	89.1
Systematic review/review	9	3.5
Cohort study	8	3.1
Case control study	6	2.3
Mixed-method and qualitative study	4	1.6
Randomized controlled trial (RCT)	1	0.4
B. Geographic region		
India	145	56.6
Bangladesh	42	16.4
Pakistan	27	10.5
Multiple countries	17	6.6
Nepal	14	5.5
Sri Lanka	10	3.9
Afghanistan	1	0.4
C. Types of data		
Primary	140	55
Secondary	116	45
D. Study theme		
TB, HIV, and other communicable Diseases	18	7
Equity and health systems	24	9
Adolescent and women health	32	13
Maternal, neonatal, and child health	98	38
Non-communicable diseases	84	33

Source of secondary data was mostly national-level household surveys such as Demographic and Health Surveys in Bangladesh (*n* = 15, 13%) and Nepal (*n* = 7, 6%) and National Family and Health Survey in India (*n* = 47 or 41%).

Thematic area wise, majority of included studies were on “maternal, neonatal, and child health (MNCH)” (*n* = 98, 38%) followed by studies on “non-communicable disease (NCD)” (*n* = 84, 33%), “adolescent and women’s health” (*n* = 32, 13%), “health systems” (*n* = 24, 9%), and studies on “TB, HIV, and other communicable disease” (*n* = 18, 7%).

Table 4 shows the distribution of SES indices used by study design, country of origin, and thematic area of studies. Majority of reviewed studies used asset-based wealth index as SES markers irrespective of study design, country of origin, or thematic area of the study. Among the cross-sectional studies, asset-based wealth index was the most commonly used SES indicator (*n* = 125, 54%), followed by SES indices based on income and expenditure (*n* = 68, 29%), indices based on occupation and education (*n =* 20, 9%), and wealth index combining education (*n* = 15, 6%). However, among the 6 case-control studies, 4 (2/3rd) used SES indices based on income and expenditure. Other study designs also mostly used asset-based wealth index. All countries used asset-based wealth index in majority of cases except Sri Lanka where majority (58%) of the studies uses SES indices based on income and expenditure. Indian studies used asset-based wealth index (*n* = 77, 52%) more than other SES indices—income and expenditure (*n* = 43, 29%), wealth index combining education (*n* = 15, 7%), and indices based on education and occupation (*n* = 10, 1%). Majority of Bangladesh studies also used asset-based wealth index (*n* = 30, 68%) followed by indices based on income and expenditure (*n* = 13, 30%). Pakistani studies used asset-based wealth index (*n* = 11, 39%) and income and expenditure (*n* = 10, 36%) as their main SES indicator. As mentioned, income and expenditure-based SES indices were used more in Sri Lankan studies (*n* = 7, 58%) than other measures of SES. Asset-based wealth index (*n* = 7, 50%) and income and expenditure-based SES (*n* = 4, 29%) were commonly used SES indices in Nepal.

**Table 4:** Distribution of different indices based on study design, country, and study theme

	Asset-based wealth index (using PCA & FA methods)	Income and expenditure	Indices based on occupation and education group	Wealth index combining education	Indices without any description
A. Study design (*n* = 265)					
Cross sectional study	125 (54%)	68 (29%)	20 (9%)	15 (6%)	5 (2%)
Systematic review/review	8 (62%)	4 (31%)	0	0	1 (8%)
Cohort study	6 (75%)	1 (13%)	0	1 (13%)	0
Case control study	1 (17%)	4 (67%)	1 (17%)	0	0
Mixed-method and qualitative study	2 (50%)	2 (50%)	0	0	0
Randomized controlled trial (RCT)	0	1 (100%)	0	0	0
B. Geographic region (*n* = 265)					
India	77 (52%)	43 (29%)	15 (10%)	10 (7%)	2 (1%)
Bangladesh	30 (68%)	13 (30%)	0	0	1 (2%)
Pakistan	11 (39%)	10 (36%)	3 (11%)	3 (11%)	1 (4%)
Multiple countries	15 (79%)	3 (10%)	0	0	1 (5%)
Nepal	7 (50%)	4 (29%)	1 (7%)	1 (7%)	1 (7%)
Sri Lanka	2 (17%)	7 (58%)	1 (8%)	2 (17%)	0
Afghanistan	1 (100%)	0	0	0	0
C. Thematic area of different studies (*n* = 256)					
Maternal, neonatal and child health	67 (66%)	23 (23%)	3 (3%)	6 (6%)	2 (2%)
Non-communicable disease (NCD)	37 (42%)	33 (37%)	11 (12%)	8 (9%)	0
Adolescent and women health	20 (63%)	9 (28%)	1 (3%)	1 (3%)	1 (3%)
Health systems	11 (46%)	6 (25%)	0	4 (17%)	3 (13%)
TB and other communicable disease	7 (37%)	9 (47%)	1 (5%)	2 (11%)	0

Thematic area-wise, MNCH-related studies used asset-based wealth index mostly (*n* = 67, 66%), followed by SES based on income and expenditure (*n* = 23, 23%) and wealth index combining education (*n* = 6, 6%). NCD-related studies used asset-based wealth index mostly (*n* = 37, 42%), followed by income and expenditure (*n* = 33, 37%), education and occupation (*n* = 11, 12%), and wealth index combing education (*n* = 8, 9%). Adolescent health studies mostly used asset-based wealth index (*n* = 20, 63%) and income and expenditure (*n* = 9, 28%) as measure of SES. Health systems-related studies also used asset-based wealth index (*n* = 11, 46%) mostly followed by indices based on income and expenditure (*n* = 6, 25%) as SES marker. Income and expenditure was the most frequently used SES indicator in TB and other communicable disease-related studies (*n* = 9, 47%) followed by asset-based wealth index (*n* = 7, 37%).

## Discussion

This scoping review is an attempt to explore the types and patterns of SES indices used in epidemiological studies conducted among South Asian urban population. The review revealed 25 different types of SES indices which can be categorized into 5 major groups. Asset-based wealth index was the mostly used SES indices in South Asian urban health studies. Uses of other SES indices were less frequent. Asset-based wealth index has been debated as the component variables are artificially constructed [14], and the method is criticized as arbitrary due to poorly defined concept of choosing variables. At the same time, its discriminating power depends on the nature and relationship of the included variables [14] which may differ in different contexts.

Almost all studies considered in this scoping review were quantitative in nature and followed cross-sectional research design mostly. There is paucity of published literature on SES measurement using data from longitudinal studies, randomized controlled trials, and qualitative and mixed-method studies. Hence, there is need for more studies of these types using SES markers. Although asset-based wealth index [5] was the most frequently used measure in describing the socioeconomic status of the target population, a number of studies used asset-based wealth index after contextualizing the study theme and study setup. Almost half of the studies were secondary analysis where different national, international, and regional survey data were used. NFHS of India [16–18] and Bangladesh Demographic and Health Survey (BDHS) [19, 20] were important among the national survey data. Indian studies used a large variety of indices where the researchers contextualized the indicator of SES with the highest frequency of asset-based wealth index use. Income-related indicators like income itself or BG Prasad’s classification [4, 21] based on income (modified several times) were as prominent as asset-based wealth index. At the same time, we observe different income category in different countries probably because of divergent currency values in India [22], Pakistan [23], Sri Lanka [24], Nepal [25], and Bangladesh [26]. Standard of living index (SLI) is calculated by adding scores on material possession following the theory of summing values of measurable quantity [27]. Total index scores ranging from 0 to 14 was considered as a low SLI, a score between 15 and 24 as a medium SLI, and a score between 25 and 67 as a high SLI [28]. Many authors used asset-based wealth index in individual context, mostly in primary studies. These contextualized indices resembles Pareekh [8], Tiwari [9], or Gour’s [10] classification. SES is estimated in Kuppuswami classification considering indicators like material possessions, highest education, highest occupation, and type of house [7, 29]. The modified Kuppuswami classification is based on occupation, education, and income which were modified in 2007 [30, 31]. Type of schooling was one of the important indices used by Indian authors in several studies [32–34] where school fees, medium of education, and type of school (public or private) were factored in the composite SES indicator. Most of the Bangladeshi studies used asset-based wealth index as the measures of SES; income was the second most frequently used indicator. Pakistani studies mostly used asset-based wealth index and “income and expenditure” based marker as SES indicator. Nepali studies used asset-based wealth index most frequently, and income was the second most used indicator. Sri Lankan studies mostly used income [24, 35] as measures of SES. Asset-based wealth index [36] and education-based indices had been applied as well. We identified only one article conducted in Afghanistan which used asset-based wealth index as measure of SES [37]. However, we did not find any study from Maldives and Bhutan in this review.

Independent of the development of new indices [38–41], most of the studies reviewed used traditional indicators such as asset-based wealth index [5] and indices based on income and expenditure. Further explorations are needed whether these common indices are capable of capturing the urban inequality properly as many urban inhabitants are transitory particularly in growing urban slums.

Majority of the studies measured health outcomes of targeted population and focused prevalence for specific diseases in population groups. Further in depth review can be considered to explore whether the researchers could satisfactorily fulfill their purpose of measuring socioeconomic status by the indices which they used. It would be useful to undertake a content analysis of the methodological and policy papers.

One of the main limitations of this review is that our analysis was limited to published scientific articles only and excluded the gray literature. This review also prioritized peer-reviewed published articles and did not include policy and institutional reports. This review provides an idea of mapping of the available indices but do not give any clarification regarding the validity and acceptability of the indices in different context, especially for the urban poor.

This review provides a detailed description of different indices used in the South Asian region. Though each of the indices has its own acceptability and limitations [27], it has been observed that some authors tried to use contextualized indices based on the population. We have found asset-based wealth index as the most frequently used index, but its acceptance is debated in the literature [3, 14, 27]. In urban setting, PCA-based approaches to designate SES is challenging due to difficulty in identifying and allocating assets [27]. Though some authors tried to use a combination of different indices, to overcome these debates, we propose further reviews of these indices against the backdrop of ever-changing nature of material wealth situation in South Asian countries.

## Conclusion

This scoping review aimed to identify the indices used to measure inequalities in health-related studies among South Asian urban population and found asset-based wealth index as the most used index. At the same time, other indices were identified which have been used in this region in different context. This review provides a distribution of all the available indices of socioeconomic status measurement. Further attempts should be made to explore the suitability of available indices to measure the socioeconomic status for the rapidly growing urban population with a transitory nature of relative wealth scenario of this region.

## Additional files

Additional file 1: Search program used for PubMed. (DOCX 11 kb)

Additional file 2: Definition of Different Indices. (DOCX 65 kb)

## Abbreviations

BDHS: Bangladesh Demographic and Health Survey
DHS: Demographic health survey
MPI: Multidimensional Poverty Index
NCDs: Non-communicable diseases
NDHS: Nepal Demographic and Health Survey
NFHS: National Family and Health Survey
PCA: Principal component analysis
SDG: Sustainable Development Goal
SES: Socioeconomic status
UBN: Unsatisfied basic needs
UHC: Universal health coverage
WI: Wealth index
