# Supplementary material for: Use of indices to measure socio-economic status (SES) in South-Asian urban health studies: a scoping review
Source: Syst Rev. 2018 Nov 17;7:196. doi: 10.1186/s13643-018-0867-6 (PMC6240202; doi:10.1186/s13643-018-0867-6)
Supplement: Supplementary file 1 — Search program used for PubMed. (DOCX 11 kb) [file 13643_2018_867_MOESM1_ESM.docx]

**Additional File 1: Search program used for PubMed**

**#1 Search: Population**

“Working Poor/economics"[Mesh] OR "Working Poor/epidemiology"[Mesh] OR "Working Poor/statistics and numerical data"[Mesh] OR "Poverty Areas"[Majr] OR urban* OR (urban* AND poor) OR ("urban poor") OR metropol* OR town* OR (Local AND Government) OR "Local authority" OR (Local AND authority)

AND

**#2 Search: Intervention**

(Wealth AND index) OR (Wealth AND quintiles) OR (Wealth AND status) OR (Wealth AND condition) OR

(Asset AND index) OR (Asset AND quintiles) OR (Asset AND status) OR (Asset AND condition) OR

(Socioeconomic AND index) OR (Socioeconomic AND quintiles) OR (Socioeconomic AND status) OR “Socioeconomic condition” OR

(Social AND index) OR (Social AND quintiles) OR (Social AND Status) OR (Social AND condition) OR

(“Socioeconomic Factors/economics"[Mesh] OR "Socioeconomic Factors/epidemiology"[Mesh] OR "Socioeconomic Factors/prevention and control"[Mesh] OR "Socioeconomic Factors/statistics and numerical data"[Mesh] OR "Socioeconomic Factors/utilization"[Mesh]) OR

“Poverty Index”

OR

“Wealth inequality” OR (Asset AND inequality) OR (“Socioeconomic inequality”) OR (“Economic inequality”)

OR

(Socioeconomic AND disparity)

AND

**#3 Search: Outcome**

Health

AND

**#4 Search: Filter**

"South Asia" OR Afghanistan OR Bangladesh OR Bhutan OR India OR Nepal OR Pakistan OR “Sri Lanka” OR Maldives
