# Supplementary material for: Use of indices to measure socio-economic status (SES) in South-Asian urban health studies: a scoping review
Source: Syst Rev. 2018 Nov 17;7:196. doi: 10.1186/s13643-018-0867-6 (PMC6240202; doi:10.1186/s13643-018-0867-6)
Supplement: Supplementary file 2 — Definition of Different Indices. (DOCX 65 kb) [file 13643_2018_867_MOESM2_ESM.docx]

**Additional File 2:**

**Definition of Different Indices**

This section highlights the definitions used to measure SES or Position. We found 25 different indexes which have been used for assessing the socioeconomic status in the included articles.

1. **Economic Status:** This index has been developed based on a grade considering the increasing rank of wealth. The rank of wealth is measured considering income and items from 20 consumer durables. This index has been categorized into Grade I, Grade II, and Grade III ([1](#_ENREF_1)). Some authors categorized the groups as Low, Middle and High on the basis of number of items counted. Persons owing zero to two items were considered as low economic status , persons owning 3–5 of the items were in middle group and high economic status was defined for persons having more than five of the items ([2](#_ENREF_2)).
2. **Expenditure:** Socioeconomic status has been indicated by expenditure. This is applicable for surveys where income data is not available. Expenditure information collected on “consumption expenditure incurred over the 30 days preceding the survey on purchase, home produced stock, receipts in exchange of goods and services, gifts and loans and free collection” which is then annualized and further converted to log values (Log Per Capita Expenditure - LPCE) ([3](#_ENREF_3)).
3. **Income:** Monthly income of family members or sometimes the head of the family is considered. Different formats like income only or per capita income ([4](#_ENREF_4)) are considered as well . Income category is different in country context as currency is different in India ([5](#_ENREF_5)), Pakistan ([6](#_ENREF_6)), Sri Lanka ([7](#_ENREF_7)), Nepal ([8](#_ENREF_8)) and Bangladesh ([9](#_ENREF_9)).
4. **Modified Kuppuswami Classification:** Socioeconomic status (SES) is estimated in Kuppuswami classification considering indicators like material possessions, highest education, highest occupation and type of house ([10](#_ENREF_10)). The Modified Kuppuswami Classification is based on Occupation, Education and Income which were modified in 2007 ([11](#_ENREF_11), [12](#_ENREF_12)). Socioeconomic class is categorized into five groups Upper, Upper Middle, Lower Middle, Upper Lower and Lower ([13](#_ENREF_13)).
5. **Social Gradient Score:** Social gradient score was derived by adding the standard of living index score with Kuppuswami classiﬁcation score as standardized for use in India ([14](#_ENREF_14)).
6. **Kutty's classification:** This socio-economic status measurement scale constructed based on assigning scores to each household on the following aspects: education, housing condition and possession of consumer durables ([15](#_ENREF_15)).
7. **Living Condition:** Living conditions was measured by merging 11 related variables. The variables are ownership of land, vegetable garden, cattle, house, number of rooms in the house, windows in the sleeping room, ventilation in kitchen, type of fuel used for cooking, distance to fuel supply, water supply at home and toilet facility at home. A score was plotted against each item resulting in a total score ranging from 0 to 11. Finally the index was categorized into three groups based on total score. Persons having a score between 0 to 4 considered as poor, a score between 5-7 was in medium status and good status was considered if the score is between 8-11([16](#_ENREF_16)).
8. **Living Index:** The Living Index was constructed by a simple count of eight household items. The items are radio/radio cassette, television, landline telephone, cellular phone, washing machine, refrigerator/freezer, CD/VCD/DVD player, and personal computer. Respondents with 4 items or less were considered as low living index, and respondents with more than 4 items were considered as a high level ([17](#_ENREF_17)).
9. **Modified BG Prasad Classification:** Prasad's classification is simply based on annual income of family members. This classification was developed in 1961([18](#_ENREF_18)) and modified in 1968 ([19](#_ENREF_19)) and 1970 by Prasad BG himself. This index was further modified in 19993-1994 by Kumar contextualizing the changing economic trends. Several modifications resulted in the latest update by Shankar Reddy in 2014 ([20](#_ENREF_20)).
10. **Multidimensional Poverty Index (MPI):** The Multidimensional Poverty Index (MPI) was initiated by the Oxford Poverty and Human Development Initiative. It considered Amartya Sen’s concept of poverty and standards of living, at the same time educational attainment and nutrition were included. Despite of its usefulness, the limitation is not to compare individual inequalities directly ([21](#_ENREF_21)). Among the dimensions and indicators, the first component reflects health considering child mortality and nutrition. The schooling years and attendance at school comprises the second component focusing education. These components were further equally weighted at 1/6. The Standard of Living built the third component with six indicators. Indicators like electricity, drinking, flooring, cooking fuel, assets like radio, tv, telephone, bike, motorbike, refrigerator and car or truck were equally weighted at 1/18 ([22](#_ENREF_22)).
11. **Occupation:** Social class was indicated on the basis of participant’s occupation. Persons with executive jobs in the government or private sectors were considered as Upper class fessionals. Non-manual workers or office job holder skilled individuals were in Middle class group. Unskilled workers like manual workers and farmers were considered as Lower class ([23](#_ENREF_23)). Some authors combined occupation and income to assess the socioeconomic position ([24](#_ENREF_24)).
12. **Occupation & Education:** In this index the modern concept of socioeconomic status considering the education and occupation of the chief wage earner of the family who contributes most was applied. By combining these variables the households were then categorized into five different classes from A–E ranging from affluent to deprived. ([25](#_ENREF_25)).
13. **Occupation, Education, Household Utility:** SES was measured based on combining the educational status, household utilities like water & electricity and occupation. Education level was given a score ranging from 0 to 2 based on no primary education, completion of primary or secondery school and higher education. The household utilities were scored based on indoor plumbing and electrical appliances. A score 0 indicated no indoor plumbing or having blender, television or refrigerator. Sore 1 was assigned for households with either one of the three items or indooor plumbing. Households with both indoor plumbing and any one of the appliances were given 2 points. Finally a five level scale was constructed ranging from 0 to 4 where a score of 0 or 1 was used to identify low-SES households ([26](#_ENREF_26)).
14. **Poverty Score:** This score is based on the World Bank’s Poverty Score Card. The score card consists of ten questions about household assets such as livestock, electronic goods, automobiles and agricultural land. Individual responses were assigned with score and a final poverty score was achieved by adding the where lower score indicates more poverty ([27](#_ENREF_27)).
15. **SES by Factor analysis:** In this index four socioeconomic factors were derived by conducting a factor analysis. Variables considered for factor analysis were child mortality rate, child/woman ratio, literacy rate, and fertility rate, source of drinking and non-drinking water, infant mortality, insolvency rate and use of solid fuels. At least three of the four derived factors being below or above the 50^th^ percentile were criteria to be categorized as either low or high SES ([28](#_ENREF_28)).
16. **Social Status Index:** The social status index composed of education, occupation, income, assets and social networking ([29](#_ENREF_29)).
17. **Socio-economic status (SES):** A composite index was constructed based on ownership of 12 household assets such as an iron, sewing machine, refrigerator, washing machine, car etc. People having up to two items were categorized as lower SES. Middle group were those having three to eight items and persons having nine or more items were in higher group ([30](#_ENREF_30)). Another way of classification is considering as low socioeconomic status who reported owning up to four items. Those who reported owning 5–9 items were considered as average socioeconomic status and those who reported owning 10 or more items were considered as upper socioeconomic status ([31](#_ENREF_31)).
18. **Socio-economic Class:** Social class was determined on the basis of paternal education and was defined as low (8 years of education or less), middle (8 to 12 years) and high (12 to 16 years or more) ([32](#_ENREF_32)).
19. **Socioeconomic Index:** Demographic, social and economic information of the families were collected to conduct a principal component analysis for generating this index. Four of these factors were considered as social such as maternal education, paternal education, maternal occupation and paternal occupation and five as economic which includes per capita income, number of rooms in the house, number of people/room, water supply and main mode of transport. The scores were then grouped to categorize families into quintiles with ‘1’ being the lowest quintile or poorest and ‘5’ as highest quintile or richest ([33](#_ENREF_33), [34](#_ENREF_34)).
20. **Socioeconomic Status Based on Education, Occupation and SE scale:** Socioeconomic status is constructed based on education, occupation and self-categorized socioeconomic scale. Socioeconomic status was identified into 10 groups depending on perceived status and grouped into high (1-3), medium (4-6) and low (7-10) ([35](#_ENREF_35)).
21. **Socioeconomic Status Scale:** For developing the scale, seven indicators namely house, materials possession, education, occupation, monthly income, land, social participation and understanding were selected. These indicators were considered as profile each of which was with five alternatives. Finally the instrument was developed on a 10 point scale ([36](#_ENREF_36), [37](#_ENREF_37)). Some authors defined it in another way. Socioeconomic status was recorded by measuring a number of variables, including level of education, income including sources, occupational status and food insecurity ([38](#_ENREF_38)).
22. **Standard Living Index:** Standard of living index (SLI), which is calculated by adding scores on following variables namely House type, Toilet facility, Source of lighting, Main fuel for cooking, Source of drinking water, Separate cooking room, Ownership different items like house, agricultural and irrigated land, livestock, durable goods such as car, motorcycle, tractor, scooter, refrigerator, pressure cooker, telephone, colour television, electric fan, bicycle, radio/transistor, mattress, cot/bed, black and white television, bullock cart, sewing machine, water pump, thresher, table, chair, clock/watch etc. Total Index scores are further categorized into three groups. A low SLI is considered when scores ranging from 0–14, a medium SLI consists of scores between 15–24 as and high SLI comprises score range 25–67 ([39](#_ENREF_39)).
23. **Types of School:** Type of schooling is considered as the measure of SES. In India, typical private schools get more students from higher economic background whereas government schools are attended by students from lower economic status ([40](#_ENREF_40)). Sometimes this indicator has been categorized as high, middle and low status considering the monthly school fees ([41](#_ENREF_41)). Often the language of school is considered as the indicator because of the higher tuition fees of the English medium schools ([42](#_ENREF_42)).
24. **Unsatisfied Basic Needs:** U.N. Economic Commission for Latin America (ECLAC) developed the framework of Unsatisfied Basic Needs (UBN) based on the works of Amartya Sen. The nonmonetary dimensions of poverty are measured through this framework using the census data independent of income. The non-income factors which are strongly associated with poverty are identified through UBN which has been adopted in different countries of Latin America ([21](#_ENREF_21)). The indicators which are used in UBN are: sources of energy used for lighting and cooking, quality of housing, employment and education of heads of household and 20 years education status of household population. ([43](#_ENREF_43)).
25. **Wealth Index:** The wealth index is developed by Rutstein (1996) and validated by Filmer and Pritchett (2001). The basic concept of wealth index is the measurement of cumulative living standard of household. The latest form of “Wealth Index” is developed by contribution from Rutstein, Gwatkin and others developed which is popularly used for demographic and health survey in many countries ([44](#_ENREF_44)). The basic indicators considered in wealth index are: ownership assets such as televisions and bicycles, housing construction materials, sanitation facilities and access to water. Using a statistical procedure known as principal components analysis (PCA), a scale of relative wealth is applied to individual households ([21](#_ENREF_21)).

***Reference***

1. Mishra SK, Mukhopadhyay S. Socioeconomic correlates of reproductive morbidity among adolescent girls in Sikkim, India. Asia-Pacific journal of public health / Asia-Pacific Academic Consortium for Public Health. 2012;24(1):136-50.

2. Zafar SN, Fatmi Z, Iqbal A, Channa R, Haider AH. Disparities in access to surgical care within a lower income country: an alarming inequity. World journal of surgery. 2013;37(7):1470-7.

3. Dutta M, Husain Z. Does health insurance ensure equitable health outcomes? An analysis of hospital services usage in urban India. World health & population. 2013;14(4):38-50.

4. Dandona R, Dandona L, John RK, McCarty CA, Rao GN. Awareness of eye diseases in an urban population in southern India. Bulletin of the World Health Organization. 2001;79(2):96-102.

5. Ramachandran A, Mary S, Yamuna A, Murugesan N, Snehalatha C. High prevalence of diabetes and cardiovascular risk factors associated with urbanization in India. Diabetes care. 2008;31(5):893-8.

6. Mushtaq MU, Majrooh MA, Ahmad W, Rizwan M, Luqman MQ, Aslam MJ, et al. Knowledge, attitudes and practices regarding tuberculosis in two districts of Punjab, Pakistan. The international journal of tuberculosis and lung disease : the official journal of the International Union against Tuberculosis and Lung Disease. 2010;14(3):303-10.

7. Rathnayake IM, Weerahewa J. Maternal employment and income affect dietary calorie adequacy in households in Sri Lanka. Food and nutrition bulletin. 2005;26(2):222-9.

8. Shrestha S, Adachi K, Petrini MA, Shrestha S. Factors associated with post-natal anxiety among primiparous mothers in Nepal. International nursing review. 2014;61(3):427-34.

9. Tarleton JL, Haque R, Mondal D, Shu J, Farr BM, Petri WA, et al. Cognitive effects of diarrhea, malnutrition, and Entamoeba histolytica infection on school age children in Dhaka, Bangladesh. The American journal of tropical medicine and hygiene. 2006;74(3):475-81.

10. Choudhary A, Moses PD, Mony P, Mathai M. Prevalence of anaemia among adolescent girls in the urban slums of Vellore, south India. Tropical doctor. 2006;36(3):167-9.

11. Kabeerdoss J, Pugazhendhi S, Subramanian V, Binder HJ, Ramakrishna BS. Exposure to hookworms in patients with Crohn's disease: a case-control study. Alimentary pharmacology & therapeutics. 2011;34(8):923-30.

12. Kajale N, Khadilkar A, Chiponkar S, Unni J, Mansukhani N. Effect of traditional food supplements on nutritional status of lactating mothers and growth of their infants. Nutrition (Burbank, Los Angeles County, Calif). 2014;30(11-12):1360-5.

13. N. Kumar CS, P. Kumar and A.S. Kundu. Kuppuswamy's Socioeconomic Status Scale-Updating for 2007. The Indian Journal of Pediatrics. 2007;74(12):1131-2.

14. Pawar AB, Mohan PV, Bansal RK. Social determinants, suboptimal health behavior, and morbidity in urban slum population: an Indian perspective. Journal of urban health : bulletin of the New York Academy of Medicine. 2008;85(4):607-18.

15. V. Raman Kutty KGB, AK. Jayasree and Jessy Thomas Prevalence of coronary heart disease in the rural population of Thiruvananthapuram district, Kerala, India. Inkmational Journal of Cardiology. 1993;39:59-70.

16. Mishra R, Hansen EH, Sabroe S, Kafle KK. Socio-economic status and adherence to tuberculosis treatment: a case-control study in a district of Nepal. International Journal of Tuberculosis and Lung Disease. 2005;9(10):1134-9.

17. Maqsood F, Flatt JD, Albert SM, Maqsood S, Nizamuddin M. Correlates of self-reported depressive symptoms: a study of older persons of Punjab, Pakistan. Journal of cross-cultural gerontology. 2013;28(1):65-74.

18. Prasad B. Social Classification of Indian families. Journal of the Indian Medical Association. 1961;37:250-1.

19. Prasad B. Social Classification of Indian families. J Indian Med Assoc 1968;51:365-66.

20. Dudala SR, KAKR, GRP. Prasad's socio-economic status classification- An update for 2014. Int J Res Health Sci. 2014;2(3):875-8.

21. Rutstein S, and Sarah Staveteig. Making the Demographic and Health Surveys Wealth Index Comparable. DHS Methodological Reports No. 9. Rockville, Maryland, USA: ICF International. 2014

22. Santos SAaME. Acute Multidimensional Poverty: A New Index for Developing Countries Oxford Poverty & Human Development Initiative (OPHI) 2010.

23. Tareen MF, Shafique K, Mirza SS, Arain ZI, Ahmad I, Vart P. Location of residence or social class, which is the stronger determinant associated with cardiovascular risk factors among Pakistani population? A cross sectional study. Rural and remote health. 2011;11(3):1700.

24. Mehta KD, Karki P, Lamsal M, Paudel IS, Majhi S, Das BK, et al. Hyperglycemia, glucose intolerance, hypertension and socioeconomic position in eastern Nepal. The Southeast Asian journal of tropical medicine and public health. 2011;42(1):197-207.

25. Turk T, Murukutla N, Gupta S, Kaur J, Mullin S, Saradhi R, et al. Using a smokeless tobacco control mass media campaign and other synergistic elements to address social inequalities in India. Cancer causes & control : CCC. 2012;23 Suppl 1:81-90.

26. van E, S L, van der H, Joshi S, Doak CM, Ponce MC. Pressure cooker ownership and food security in Aurangabad, India. Public health nutrition. 2012;15(5):818-26.

27. Leon SIGaDA. Prevalence and sociodemographic determinants of tobacco use among adults in Pakistan: findings of a nationwide survey conducted in 2012. Population Health Metrics 2013;11(16).

28. Burkart K, Khan Mobarak H, Kraemer A, Breitner S, Schneider A, Endlicher Wilfried R. Seasonal variations of all-cause and cause-specific mortality by age, gender, and socioeconomic condition in urban and rural areas of Bangladesh. International journal for equity in health. 2011;10.

29. De S, Ambepitiyawaduge P, De S, Sudirikku Hennadige P, Haniffa R, Liyanage Isurujith K, et al. A survey on socioeconomic determinants of diabetes mellitus management in a lower middle income setting. International journal for equity in health. 2016;15.

30. Fikree FF, Ali TS, Durocher JM, Rahbar MH. Newborn care practices in low socioeconomic settlements of Karachi, Pakistan. Social science & medicine (1982). 2005;60(5):911-21.

31. Fariyal F Fikree AK, Muhammad Masood Kadir, Fatima Sajan, Mohammad H. Rahbar. What influences contraceptive use among young women in Urban squatter settlements of Karachi, Pakistan. International family planning perspectives. 2001;27(3):130-6.

32. Sughis M, Nawrot TS, Ihsan-ul-Haque S, Amjad A, Nemery B. Blood pressure and particulate air pollution in schoolchildren of Lahore, Pakistan. BMC public health. 2012;12:378.

33. Avan Bilal I, Kirkwood B. Role of neighbourhoods in child growth and development: Does 'place' matter? Social Science & Medicine. 2010;71(1):102-9.

34. Avan BI, Raza SA, Kirkwood BR. A community-based study of early childhood sensory stimulation in home environment associated with growth and psychomotor development in Pakistan. International journal of public health. 2014;59(5):779-88.

35. Gupta R, Deedwania Prakash C, Sharma K, Gupta A, Guptha S, Achari V, et al. Association of Educational, Occupational and Socioeconomic Status with Cardiovascular Risk Factors in Asian Indians: A Cross-Sectional Study. PloS one. 2012;7(8).

36. Tiwari SC, Kumar A, Kumar A. Development & standardization of a scale to measure socio-economic status in urban & rural communities in India. The Indian journal of medical research. 2005;122(4):309-14.

37. Aggarwal AN, Gupta D, Janmeja AK, Jindal SK. Assessment of health-related quality of life in patients with pulmonary tuberculosis under programme conditions. The international journal of tuberculosis and lung disease : the official journal of the International Union against Tuberculosis and Lung Disease. 2013;17(7):947-53.

38. Prina AM, Ferri CP, Guerra M, Brayne C, Prince M. Prevalence of anxiety and its correlates among older adults in Latin America, India and China: cross-cultural study. The British journal of psychiatry : the journal of mental science. 2011;199(6):485-91.

39. Macro IIfPSIaO. National Family Health Survey (NFHS-2), 1998–99: India. Mumbai: IIPS. 2000.

40. Mathur C, Stigler MH, Erickson DJ, Perry CL, Finnegan JR, Jr, et al. Socioeconomic differences in the risk profiles of susceptibility and ever use of tobacco among Indian urban youth: a latent class approach. Nicotine & tobacco research : official journal of the Society for Research on Nicotine and Tobacco. 2014;16(4):454-60.

41. Mushtaq Muhammad U, Gull S, Shahid U, Shafique Mahar M, Abdullah Hussain M, Shad Mushtaq A, et al. Family-based factors associated with overweight and obesity among Pakistani primary school children. BMC pediatrics. 2011;11.

42. Pauline M, Selvam S, Swaminathan S, Vaz M. Body weight perception is associated with socio-economic status and current body weight in selected urban and rural South Indian school-going children. Public health nutrition. 2012;15(12):2348-56.

43. Satharasinghe A. Census Dept. Classifies GN Divisions by Poverty Department of census and statistics, Colombo. 2008.

44. Rutstein SOaKJ. The DHS Wealth Index. DHS Comparative Reports No. 6. . Calverton, Maryland: ORC Macro. 2004.
